# Work Impairment and Financial Outcomes Among Adults With vs Without Long COVID

**DOI:** 10.1001/jamanetworkopen.2025.26310

**Published:** 2025-08-12

**Authors:** Michael Gottlieb, Ji Chen, Huihui Yu, Michelle Santangelo, Erica S. Spatz, Nicole L. Gentile, Rachel E. Geyer, Caitlin Malicki, Kristyn Gatling, Kelli N. O’Laughlin, Kari A. Stephens, Joann G. Elmore, Lauren E. Wisk, Michelle L’Hommedieu, Robert Rodriguez, Juan Carlos C. Montoy, Ralph C. Wang, Kristin L. Rising, Efrat Kean, Jonathan W. Dyal, Mandy J. Hill, Arjun K. Venkatesh, Robert A. Weinstein

**Affiliations:** 1 Department of Emergency Medicine, Rush University Medical Center, Chicago, Illinois; 2 Section of Cardiovascular Medicine, Yale School of Medicine, New Haven, Connecticut; 3 Yale Center for Outcomes Research and Evaluation, New Haven, Connecticut; 4 Department of Epidemiology, Yale School of Public Health, New Haven, Connecticut; 5 Department of Family Medicine, University of Washington, Seattle; 6 Department of Laboratory Medicine and Pathology, University of Washington, Seattle; 7 Department of Rehabilitation Medicine, University of Washington, Seattle; 8 Department of Emergency Medicine, Yale School of Medicine, New Haven, Connecticut; 9 Division of Infectious Diseases, Department of Medicine, Rush University Medical Center, Chicago, Illinois; 10 Department of Emergency Medicine, University of Washington, Seattle; 11 Department of Global Health, University of Washington, Seattle; 12 Biomedical Informatics & Medical Education, University of Washington, Seattle; 13 Division of General Internal Medicine & Health Services Research, David Geffen School of Medicine at UCLA, University of California, Los Angeles; 14 UCLA National Clinician Scholar Program, Los Angeles, California; 15 Department of Emergency Medicine, University of California, San Francisco; 16 Department of Emergency Medicine, Sidney Kimmel Medical College, Philadelphia, Pennsylvania; 17 Center for Connected Care, Thomas Jefferson University, Philadelphia, Pennsylvania; 18 Department of Emergency Medicine, Johns Hopkins University School of Medicine, Baltimore, Maryland; 19 Department of Population Health and Health Disparities, School of Public and Population Health at the University of Texas Medical Branch at Galveston, Galveston; 20 Center for Outcomes Research and Evaluation, Yale New Haven Hospital, New Haven, Connecticut; 21 Rush University Medical Center, Chicago, Illinois; 22 Cook County Hospital, Chicago, Illinois; 23 The CORE Center, Chicago, Illinois

## Abstract

**Importance:**

While much of the focus on long COVID (LC; defined as developing new, persistent symptoms lasting 3 months or longer after SARS-CoV-2 infection) has been on health status and quality of life, the impact on individual work productivity and financial distress are less well established.

**Objectives:**

To assess differences in work and financial outcomes among individuals with current, resolved, and no LC up to 3 years after initial infection.

**Design, Setting, and Participants:**

This prospective, multisite, longitudinal cohort study enrolled adult participants (age ≥18 years) with at least 1 reported SARS-CoV-2 infection from December 7, 2020, to August 29, 2022. Follow-up electronic surveys were collected through April 2, 2024. Data were analyzed from January 20 to February 4, 2025.

**Exposure:**

Self-reported resolved or current LC and vaccination status.

**Main Outcomes and Measures:**

Financial toxicity was measured using Comprehensive Score for Financial Toxicity–Functional Assessment of Chronic Illness Therapy (FACIT-COST), and work impact was measured using the Work Productivity & Activity Impairment questionnaire (version 2.0).

**Results:**

Of 3663 participants (mean [SD] age, 40.2 [14.2] years; 2429 [66.3%] female), 994 (27.1%) reported current LC, 2604 (71.1%) never had LC, and 65 (1.8%) had resolved LC. Participants with current LC reported more overall work impairment due to health (mean [SD], 17.7% [25.3%] of total hours worked per week) compared with those who never had LC (mean [SD], 3.2% [11.8%] of total hours) and resolved LC (mean [SD], 5.6% [12.2%] of total hours), with significantly increased odds of any work impairment compared with those who never had LC (adjusted odds ratio [aOR], 7.24; 95% CI, 5.68-9.21). The current LC group had increased odds of missing work due to their health (aOR, 2.62; 95% CI, 1.93-3.57) and of experiencing work impairment (aOR, 11.82; 95% CI, 8.90-15.70) compared with the group who never had LC. Individuals with current LC had increased odds of having moderate to high FACIT-COST scores compared with those who never had LC (aOR, 5.20; 95% CI, 3.92-6.89) and compared with those with resolved LC (aOR, 3.16; 95% CI, 1.19-8.41). Participants who were vaccinated had lower overall work impairment (aOR, 0.71; 95% CI, 0.55-0.92), impairment while working (aOR, 0.66; 95% CI, 0.50-0.87), impairment of nonwork activities (aOR, 0.74; 95% CI, 0.57-0.96), and financial toxicity (least-squares mean difference, 1.07; 95% CI, 0.19-1.95) compared with those who were not vaccinated.

**Conclusions and Relevance:**

In this prospective cohort study of adults with SARS-CoV-2 infection, participants with current self-reported LC reported worse work impairment, missed work, and financial distress compared with those who never had LC, while vaccination was associated with improved work outcomes and less financial distress even among individuals with LC. These data underscore the need for postpandemic assistance programs, as well as vaccination to decrease societal harms.

## Key Points

**Question:**

What are the differences in long-term financial and work outcomes among individuals with self-reported long COVID (LC) vs those with resolved or never LC?

**Findings:**

In this cohort study of 3663 participants, financial and work outcomes were worse in participants with current LC up to 3 years after initial infection. COVID-19 vaccination was associated with better work and financial outcomes.

**Meaning:**

This cohort study found that worse financial outcomes associated with LC were sustained up to 3 years after SARS-CoV-2 infection but were mitigated by prior vaccination.

## Teaser

This cohort study describes differences in work and financial outcomes among individuals with current, resolved, and no LC up to 3 years after initial infection

## Introduction

There have been more than 777 million reported cases of COVID-19 worldwide.^1^ Among individuals with COVID-19, approximately 13% will develop new persistent symptoms lasting 3 months or longer, a condition commonly referred to as *long COVID* (LC).^2^ Symptoms of LC can be myriad, with the most common including fatigue, dyspnea, concentration, and memory issues.^3^ These can have a profound impact on quality of life, with markedly worse outcomes among individuals with LC compared with those without.^4^

While most prior LC research has understandably focused on health status and health care–related quality-of-life outcomes, the impact of LC on individual work productivity and the financial toxicity of LC are less characterized. The macroeconomic effects of the pandemic are well established, while the individual financial toll and impact on return to work remain underexplored. Early research suggested that rates of return to work among individuals with persistent LC varied widely, ranging from 10% to 100%.^5^ Many of these studies were limited by small sample sizes, short follow-up periods, and high percentages of participants experiencing prolonged hospitalizations or intensive care unit stays. A 2024 meta-analysis reported that approximately 60% of individuals with LC returned to work by 12 weeks, with many experiencing work restrictions.^6^ However, there remained marked heterogeneity across studies with regard to time period and study populations, and many studies did not use validated tools for assessing return to work or work productivity. Moreover, these studies primarily took place in Europe, with no studies in the meta-analysis conducted in the US. Given the unique work culture in the US and a population exceeding 340 million people, it is imperative to better understand the impact of LC on return-to-work in this setting. Additionally, given debates in society around benefits of vaccination, defining the impact of LC on individual return-to-work is critical to inform such debates.

Considering the substantial number of people impacted by LC, it is also important to understand the financial impact of LC at an individual level. One study of LC among people in the United Kingdom reported worse subjective ratings of financial well-being in individuals with LC.^7^ Another study reported an association with reductions in overall income.^8^ However, much of the prior research has focused solely on income or has used subjective tools, with limited understanding of the broader financial impact assessed by more comprehensive and validated tools. Financial toxicity provide a more robust measure to report the economic burden of medical care for patients that can impact well-being and quality of life.^9^ To address these gaps, we sought to use data from the Innovative Support for Patients with SARS-CoV-2 Infections Registry (INSPIRE) study to analyze return to work, work productivity, and financial toxicity among individuals with current LC, resolved LC, and no LC.

## Methods

This cohort study received institutional review board approval at all participating institutions. All participants provided written informed consent. This study was prospectively registered on ClinicalTrials.gov (identifier: NCT04610515). The study adhered to the Strengthening the Reporting of Observational Studies in Epidemiology (STROBE) reporting guideline.^10^

### Study Design

INSPIRE is a prospective, longitudinal study conducted across 8 major health care institutions in the US that were selected for diversity of geographic location and participant populations (eAppendix in Supplement 1).^11^ The initial study cohort included 6044 US adults with COVID-19–like symptoms, regardless of SARS-CoV-2 test results, who enrolled in person or virtually between December 7, 2020, and August 29, 2022, and were followed-up through March 15, 2023. Inclusion criteria were age at least 18 years, fluency in English or Spanish, self-reported symptoms suggestive of SARS-CoV-2 (eg, fever, cough) at the time of testing, and testing with a molecular- or antigen-based assay approved by the US Food and Drug Administration within the preceding 42 days. Exclusion criteria have been well described elsewhere^11^ and included inability to provide consent, being lawfully imprisoned, inability of the study team to confirm the result of the index diagnostic test for SARS-CoV-2, having a previous SARS-CoV-2 infection more than 42 days before enrollment, and lacking access to an internet-connected device (eg, smartphone, tablet, computer) for electronic survey completion. Recruitment of the original INSPIRE cohort was completed broadly without geographic or health system limitations.

Original study activities included completion of electronic surveys (baseline, quarterly, and optional final survey) and sharing of electronic medical records via a patient-portal (required through March 21, 2022). Eligible participants were offered a consent addendum for a long-term follow-up survey, responses from which were used in this analysis. Eligible participants included those who were not withdrawn or deceased at the end of the original study and did not opt out of study extension communications. Long-term surveys were completed from February 27, 2024, to April 2, 2024, which was 18 to 40 months after index SARS-CoV-2 infection. Participants had 28 days to complete the survey after completing the consent addendum and received $100 for survey completion. Surveys were collected via REDCap software (Vanderbilt) and sent via email or text, based on participant preference.^12,13^

This analysis included INSPIRE participants who completed the consent addendum and long-term survey. To enable comparisons of participants with and without LC, we restricted our cohort to those who reported at least 1 SARS-CoV-2 infection on the long-term survey.

### Study Measures

Study data were collected in the long-term survey, except for demographics (eg, age, gender, race, ethnicity), which were collected on the baseline survey at initial study enrollment. Race was self-reported and categorized as Asian, Black or African American, White, and other (eg, American Indian, Alaskan Native, Native Hawaiian, Other Pacific Islander, or other race not specified) or multiple races, and ethnicity was categorized as Hispanic or not Hispanic. Age, gender, race, and ethnicity were assessed to account for potential confounding by societal factors.

LC status was determined by the following question: “Following COVID-19 infections, some people may develop a condition called Long COVID. This is defined as having symptoms (such as fatigue, shortness of breath, brain fog, etc.) that last for more than 12 weeks or having symptoms that suddenly emerge without another explanation. This condition is called Long COVID. Do you think you have had Long COVID?” with options to respond yes or no. Participants responding yes were provided with a list of previously entered dates of SARS-CoV-2 infections (month and year) and asked to select after which infection their LC symptoms first began. They were then asked, “Since the start of your Long COVID symptoms, how have your symptoms changed over time?” with response options of “My symptoms have 1) improved, 2) gotten worse, 3) not changed, 4) waxed and waned (shifted back and forth), and 5) fully resolved.” Participants reporting no to having LC were assigned to the never had LC group, those reporting fully resolved symptoms were assigned to the resolved LC group, and the remainder were assigned to the current LC group. Among participants with either current or resolved LC, LC duration was calculated by the difference in months between LC onset (primary infection after which LC symptoms began) and survey completion date. Participants with current LC were assigned to a symptom evolution category (improved, worsened, no change, waxed and waned) based on survey responses. We intentionally used self-report of LC to be consistent with the more recent approaches, which emphasize the multitude of potential symptoms and important role of patient involvement in defining LC.^14^

The survey tool was developed by study investigators with specific feedback from a patient advisory board and informed by the literature. The patient advisory board reviewed the items and provided focused feedback to establish content and response process validity. Survey items included questions regarding participants’ current work status and the impact of LC on their work experience.

In addition, we quantified the degree of missed work and work impairment using the Work Productivity and Activity Impairment Questionnaire (WPAI) version 2.0, a validated 6-item tool for assessing the degree of impairment in work due to chronic illness.^15,16^ The WPAI responses were then used to calculate 4 outcomes: (1) percentage of total work hours per week that were missed due to health, (2) percentage of total work hours per week that an individual worked while impaired due to health, (3) percentage overall work impairment (which includes both missed work and work while impaired), and (4) percentage of total hours per week that an individual experienced non–work-related activity impairment.

We evaluated the degree of financial toxicity using Functional Assessment of Chronic Illness Therapy Comprehensive Score for Financial Toxicity (FACIT-COST), a validated tool for assessing financial impact of chronic illness among patients with cancer that was adapted for LC. FACIT-COST is an 11-item questionnaire scored on a 5-point scale, with lower scores indicating with worse financial toxicity.^17^

### Statistical Analysis

We analyzed differences in participant demographics, financial outcomes, and work impairment across LC status groups. Categorical variables were compared using χ^2^ or Fisher exact tests, while continuous variables were assessed using the Kruskal-Wallis tests.

To evaluate the association of LC status with financial toxicity and work impairment, we selected statistical models based on the distribution of each outcome. For WPAI outcomes, the analysis was restricted to participants who were currently employed, as only they were required to complete the questionnaire. Because there was a large number of respondents with a score of zero on the WPAI, indicating no work impairment, we applied a 2-part model using a zero-inflated β regression^18,19^: (1) the zero-inflation part used logistic regression to estimate the odds of experiencing any work impairment (WPAI outcome >0) and (2) the nonzero part used β regression with logit link to assess the odds of having a higher percentage of work impairment among participants with WPAI greater than 0. Adjusted odds ratios (aORs) were reported for both parts. For the FACIT-COST score, we used linear regression models to assess differences in continuous scores, reporting adjusted least-squares (LS) mean differences as measures of effect size for resolved and current LC. In addition, we conducted a logistic regression analysis to estimate the odds of experiencing moderate to high financial toxicity (score <14),^20^ and aORs were reported.

Each outcome model was run in both an unadjusted version (including only LC status) and an adjusted version, adjusting for age, gender, race, and ethnicity (eFigures 1-3 in Supplement 1). Furthermore, we adjusted for SARS-CoV-2 vaccination status before the initial infection to assess its overall association and reported adjusted LS mean differences and aORs comparing participants vaccinated and not vaccinated before the initial infection for each outcome.

We used SAS version 9.4 (SAS Institute) and R version 4.3.3 (R Project for Statistical Computing) for statistical analyses and Excel version 16.98 (Microsoft) for visualization. Given the exploratory nature of this study, no multiplicity adjustments were performed. All tests were 2-sided with a significance threshold of *P* < .05. Data were analyzed from January 20 to February 4, 2025.

## Results

**Figure 1.**
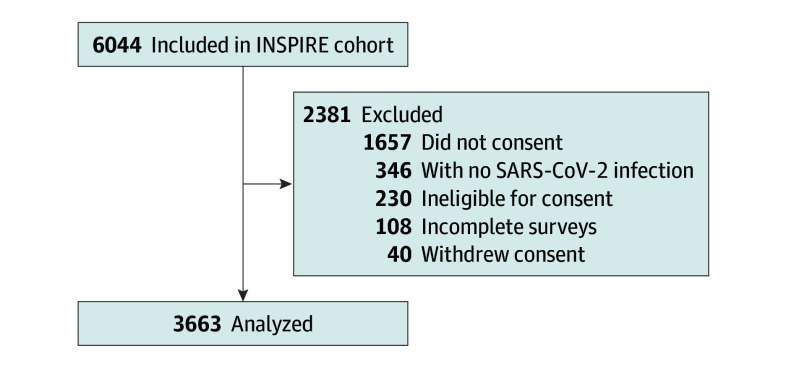
Participant Enrollment Flowchart INSPIRE indicates Innovative Support for Patients with SARS-CoV-2 Infections Registry.

Among 4119 INSPIRE participants who consented to receive the long-term survey, 4009 completed the survey, of whom 3663 (91.4%) reported at least 1 SARS-CoV-2 infection since enrollment and qualified for analysis (Figure 1). The mean (SD) age was 40.2 (14.2) years and 2429 (66.3%) were female. Overall, 510 participants (13.9%) were Hispanic or Latino ethnicity and 3082 participants (84.1%) were not Hispanic or Latino; there were 499 Asian participants (13.6%), 281 Black or African American participants (7.7%), 2438 White participants (66.6%), and 335 participants (9.1%) self-identified as another or multiple races. Full demographics by LC status are included in Table 1. In total, 2604 participants (71.1%) reported never having LC, 994 participants (27.1%) had current LC, and 65 participants (1.8%) had resolved LC. Among participants with current LC, 467 participants (47.0%) reported symptoms that waxed and waned, 258 participants (26.0%) reported improved symptoms, 187 participants (18.8%) reported no change, and 82 participants (8.2%) reported worsened symptoms. Among participants with current LC, 153 participants (15.4%) were vaccinated prior to LC onset. Nearly all of the study participants (3482 [95.1%]) received at least 1 COVID-19 vaccine, with most participants (2609 [71.2%]) reporting having received 3 to 5 doses of a COVID-19 vaccine.

**Table 1.** Demographic Characteristics by Self-Reported LC Status

Characteristic	Participants, No. (%)				*P* value^a^
	Total (N = 3663)	LC			
		Never (n = 2604)	Resolved (n = 65)	Current (n = 994)	
Age, y					
Mean (SD)	40.2 (14.2)	39.6 (14.3)	37.1 (13.8)	41.9 (13.9)	<.001
18 to 34	1532 (41.8)	1156 (44.4)	33 (50.8)	343 (34.5)	<.001
35 to 49	1171 (32.0)	791 (30.4)	19 (29.2)	361 (36.3)	
50 to 64	659 (18.0)	440 (16.9)	9 (13.8)	210 (21.1)	
≥65	274 (7.5)	193 (7.4)	3 (4.6)	78 (7.8)	
Missing	27 (0.7)	24 (0.9)	1 (1.5)	2 (0.2)	
Gender					
Female	2429 (66.3)	1663 (63.9)	43 (66.2)	723 (72.7)	<.001
Male	1067 (29.1)	828 (31.8)	17 (26.2)	222 (22.3)	
Transgender, nonbinary, or other	62 (1.7)	33 (1.3)	3 (4.6)	26 (2.6)	
Missing	105 (2.9)	80 (3.1)	2 (3.1)	23 (2.3)	
Ethnicity					
Hispanic	510 (13.9)	316 (12.1)	14 (21.5)	180 (18.1)	<.001
Non-Hispanic/Latino	3082 (84.1)	2235 (85.8)	50 (76.9)	797 (80.2)	
Missing	71 (1.9)	53 (2.0)	1 (1.5)	17 (1.7)	
Race					
Asian	499 (13.6)	388 (14.9)	12 (18.5)	99 (10.0)	<.001
Black or African American	281 (7.7)	173 (6.6)	9 (13.8)	99 (10.0)	
White	2438 (66.6)	1758 (67.5)	35 (53.8)	645 (64.9)	
Other or multiple races^b^	335 (9.1)	214 (8.2)	4 (6.2)	117 (11.8)	
Missing	110 (3.0)	71 (2.7)	5 (7.7)	34 (3.4)	
Essential worker					
Yes	1244 (41.6)	887 (41.0)	14 (29.8)	343 (44.1)	.02
No	1708 (57.1)	1257 (58.1)	33 (70.2)	418 (53.8)	
Missing	37 (1.2)	21 (1.0)	0 (0.0)	16 (2.1)	
Vaccination status before initial infection					
Not vaccinated	608 (21.4)	390 (19.6)	8 (15.4)	210 (26.2)	<.001
Vaccinated	2231 (78.6)	1596 (80.4)	44 (84.6)	591 (73.8)	

Abbreviation: LC, long COVID.

^a^ The Kruskal-Wallis test for continuous variable and χ^2^ tests for categorical variables were conducted to obtain the *P* values.

^b^ Other includes American Indian, Alaskan Native, Native Hawaiian, Other Pacific Islander, or other race not specified.

At the time of the long-term survey completion, 674 participants (18.4%) were not currently employed, with a higher rate among those with resolved LC (18 participants [27.7%]) or current LC (217 participants [21.8%]) vs never-having LC (439 participants [16.9%]) (Table 2). A higher proportion of participants with current LC reported having reduced work hours (256 participants [25.8%]) compared with resolved LC (10 participants [15.4%]) or no LC (352 participants [13.5%]). Participants with current LC also were more likely to experience periods of unemployment (221 participants [22.2%]) compared with resolved LC (12 participants [18.5%]) or no LC (367 participants [14.1%]). Most participants with current LC attributed the work loss as a direct result of their symptoms (243 participants [59.0%]). Work status requests had been made by 237 participants (23.8%) with current LC, and included reduced work hours, extended periods of time off, transition to remote or hybrid work, or requiring disability services.

**Table 2.** Financial and Work Outcomes by Self-Reported LC Status

Outcome	Participants, No. (%)				*P* value^a^
	Total (N = 3663)	LC			
		Never (n = 2604)	Resolved (n = 65)	Current (n = 994)	
Currently employed					
Yes	2989 (81.6)	2165 (83.1)	47 (72.3)	777 (78.2)	<.001
No	674 (18.4)	439 (16.9)	18 (27.7)	217 (21.8)	
Work status (if employed)					
Full-time (≥30 h/wk)	2441 (81.7)	1780 (82.2)	36 (76.6)	625 (80.4)	.10
Part-time (<30 h/wk)	405 (13.5)	294 (13.6)	6 (12.8)	105 (13.5)	
Self-employed	143 (4.8)	91 (4.2)	5 (10.6)	47 (6.0)	
Any loss of work since the start of the COVID-19 pandemic					
Reduced work hours	618 (16.9)	352 (13.5)	10 (15.4)	256 (25.8)	<.001
Unemployment	600 (16.4)	367 (14.1)	12 (18.5)	221 (22.2)	<.001
None of the above	2596 (70.9)	1970 (75.7)	44 (67.7)	582 (58.6)	<.001
Work loss as a direct result of a SARS-CoV-2 infection or ongoing symptoms					
Yes	443 (41.7)	192 (30.5)	8 (38.1)	243 (59.0)	<.001
No	620 (58.3)	438 (69.5)	13 (61.9)	169 (41.0)	
Requested a change or adjustment in work status as a result of a SARS-CoV-2 infection or ongoing symptoms					
Yes	485 (13.2)	237 (9.1)	11 (16.9)	237 (23.8)	<.001
No	3178 (86.8)	2367 (90.9)	54 (83.1)	757 (76.2)	
Work status request (if requested)					
Reduced work hours	174 (4.8)	69 (2.6)	7 (10.8)	98 (9.9)	<.001
Extended period of time-off	213 (5.8)	94 (3.6)	5 (7.7)	114 (11.5)	<.001
Remote work or hybrid	261 (7.1)	139 (5.3)	5 (7.7)	117 (11.8)	<.001
Disability benefits	34 (0.9)	4 (0.2)	1 (1.5)	29 (2.9)	<.001
Other	22 (0.6)	12 (0.5)	0	10 (1.0)	.14
Financial toxicity					
FACIT-COST score, Mean (SD)	28.8 (10.2)	31.1 (8.8)	28.2 (9.7)	22.7 (10.9)	<.001
Any					
Absent (FACIT-COST score ≥26)	2440 (66.6)	1981 (76.1)	38 (58.5)	421 (42.4)	<.001
Present (FACIT-COST score <26)	1223 (33.4)	623 (23.9)	27 (41.5)	573 (57.6)	
Severity					
None/mild (FACIT-COST score ≥14)	3320 (90.6)	2483 (95.4)	60 (92.3)	777 (78.2)	<.001
Moderate/high (FACIT-COST score <14)	343 (9.4)	121 (4.6)	5 (7.7)	217 (21.8)	
WPAI					
Work time missed due to health					
Overall, mean (SD), %	3.2 (13.6)	2.1 (10.7)	2.7 (9.3)	6.2 (19.4)	<.001
Any [≥1%]	243 (8.8)	123 (6.2)	5 (11.6)	115 (16.0)	<.001
Subgroup with ≥1%, mean (SD), %	35.9 (30.4)	33.7 (28.4)	23.2 (17.5)	38.9 (32.7)	.27
Impairment while working due to health					
Overall, mean (SD), %	5.3 (15.0)	1.9 (9.0)	3.0 (8.0)	15.0 (22.8)	<.001
Any [≥1%]	424 (15.6)	124 (6.3)	7 (16.3)	293 (41.8)	<.001
Subgroup with ≥1%, mean (SD), %	33.8 (22.0)	29.8 (21.3)	18.6 (10.7)	35.9 (22.2)	.006
Overall work impairment due to health					
Overall, mean (SD), %	7.0 (17.6)	3.2 (11.8)	5.6 (12.2)	17.7 (25.3)	<.001
Any [≥1%]	541 (19.9)	210 (10.6)	10 (23.3)	321 (45.8)	<.001
Subgroup with ≥1%, mean (SD), %	35.1 (23.8)	30.1 (22.4)	24.1 (14.0)	38.6 (24.3)	<.001
Activity impairment due to health					
Overall, mean (SD), %	6.0 (16.7)	2.4 (10.4)	1.7 (4.8)	16.4 (24.9)	<.001
Any [≥1%]	500 (16.8)	170 (7.9)	6 (12.8)	324 (41.7)	<.001
Subgroup with ≥1%, mean (SD), %	36.0 (24.1)	30.3 (22.9)	13.3 (5.2)	39.4 (24.2)	<.001

Abbreviations: LC, long COVID; WPAI, work productivity and activity impairment questionnaire.

^a^ The Kruskal-Wallis test for continuous variable and χ^2^ tests for categorical variables were conducted to obtain the *P* values.

**Figure 2.**
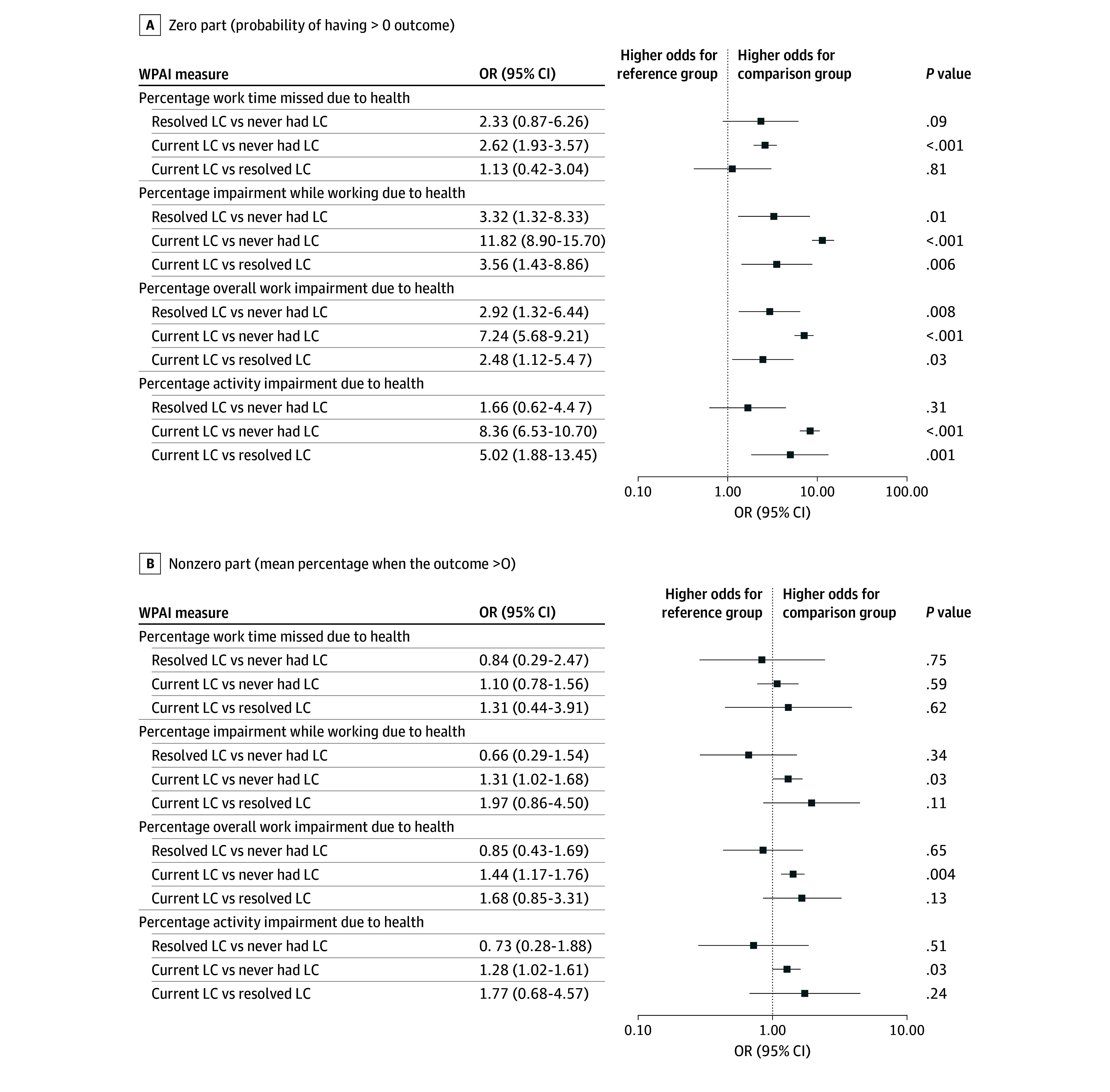
Differences in Work Productivity Among Individuals With Current, Resolved, and Never Long COVID (LC) OR indicates odds ratio, WPAI, Work Productivity and Activity Impairment Questionnaire.

We assessed the productivity of participants who were currently employed using the WPAI tool. The current LC cohort experienced a greater overall percentage of work impairment due to health conditions (reflecting a mean [SD] of 17.7% [25.3%] of total hours worked per week) compared with the resolved LC cohort (mean [SD], 5.6% [12.2%] of total hours per week) and no LC cohort (mean [SD], 3.2% [11.8%] of total hours per week). Compared with individuals without LC, the current LC cohort had significantly higher odds of experiencing any overall work impairment due to their health (aOR, 7.24; 95% CI, 5.68-9.21). Among individuals who reported any impairment, the current LC cohort also had higher odds of impairment compared with those without LC (aOR, 1.44; 95% CI, 1.17-1.76) (Figure 2).

When analyzed for missed work only, the current LC cohort missed a mean (SD) 6.2% (19.4%) of total scheduled hours compared with 2.7% (9.3%) in the resolved LC and 2.1% (10.7%) in the no LC cohort. Compared with individuals without LC, those with current LC had significantly higher odds of missing any work due to health (aOR, 2.62; 95% CI, 1.93-3.57). Among those who missed any work, there was no statistically significant difference in work missed (aOR, 1.10; 95% CI, 0.78-1.56). When analyzed by impairment at work, participants in the current LC cohort reported higher proportions of impairment at work due to health (reflecting a mean [SD] of 15.0% [22.8%] of total worked hours) compared with those with resolved LC (mean [SD], 3.0% [8.0%] of total worked hours) or no LC (mean [SD], 1.9% [9.0%] of total worked hours). Compared with individuals without LC, those with current LC had significantly higher odds of experiencing any work impairment due to health (aOR, 11.82; 95% CI, 8.90-15.70). Among those who experienced any impairment, the current LC cohort also had higher odds of impairment compared with those without LC (aOR, 1.31; 95% CI, 1.02-1.68).

For nonwork activities, the current LC cohort experienced a higher percentage of impairment (mean [SD], 16.4% [24.9%] of total hours) compared with resolved LC (mean [SD], 1.7% [4.8%] of total hours) or no LC (mean [SD], 2.4% [10.4%] of total hours). Compared with individuals without LC, those with current LC had significantly higher odds of experiencing any activity impairment (aOR, 8.36; 95% CI, 6.53-10.70). Among those who experienced any impairment, the current LC cohort also had higher odds of impairment compared with those without LC (aOR, 1.28; 95% CI, 1.02-1.61).

**Figure 3.**
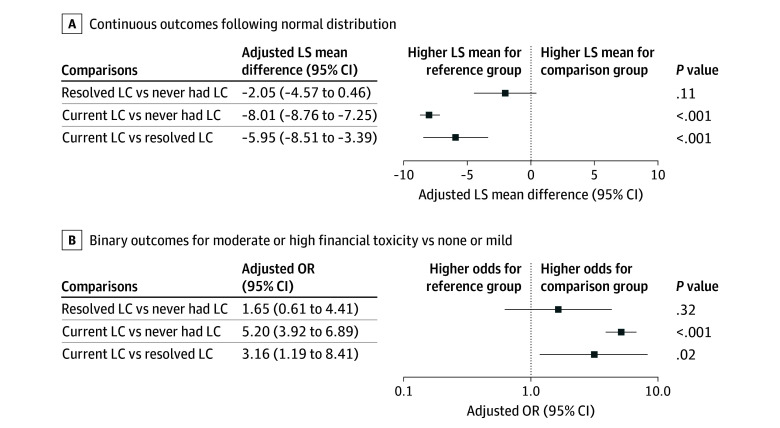
Difference in Financial Toxicity Among Individuals With Current, Resolved, and Never Long COVID (LC) LS indicates least-squares; OR, odds ratio.

Participants with current LC had numerically lower (ie, worse) mean FACIT-COST scores compared with never having LC (LS mean difference, −8.01; 95% CI, −8.76 to −7.25) or resolved LC (LS mean difference, −5.95; 95% CI, −8.51 to −3.39) (Figure 3A). When analyzed by severity of financial toxicity, the current LC group had higher odds of having moderate to high financial toxicity vs with the no LC cohort (aOR, 5.20; 95% CI, 3.92 to 6.89) and the resolved LC cohort (aOR, 3.16; 95% CI, 1.19 to 8.41) (Figure 3B).

When analyzed by vaccination status, those who were vaccinated had lower odds of overall work impairment (aOR, 0.71; 95% CI, 0.55-0.92), impairment while working (aOR, 0.66; 95% CI, 0.50-0.87), and impairment of nonwork activities (aOR, 0.74; 95% CI, 0.57-0.96) (eTable 1 in Supplement 1). Among those vaccinated, there was a lower FACIT-COST score (adjusted LS mean difference, 1.07; 95% CI, 0.19-1.95) but no significant difference in severity of financial toxicity (aOR, 0.77; 95% CI, 0.57-1.04). Findings by age, race, ethnicity, and gender are provided in the eResults in Supplement 1.

## Discussion

The findings of this cohort study highlight the substantial association of LC with employment and financial well-being extending up to 3 years after initial SARS-CoV-2 infection. Among 3663 participants, approximately one-quarter reported ongoing symptoms of LC, with higher odds of experiencing worse rates of returning to full-time work and worse overall productivity, general activity impairment, and financial outcomes compared with those who never had LC or with resolved LC. These results underscore the persistent and debilitating nature of LC.

Participants with current LC reported important employment challenges compared with those who never had LC. The current LC cohort had higher rates of participants who were not employed or had reduced work hours, combining to demonstrate that nearly half of all participants with current LC had not returned to full-time work by up to 3 years later. This is consistent with prior research demonstrating lower employment among individuals with LC and adds valuable data about the US population and the sustained effects after initial infection.^5,6^

Using the WPAI tool, individuals with current LC who were working demonstrated significantly higher overall impairment in their ability to work compared with those with resolved LC or who never had LC. The current LC group experienced overall work impairment affecting nearly one-quarter (22.1%) of their total hours, meaning impairment of more than 1 day per workweek on average due to LC. Furthermore, participants with current LC missed more work hours and reported greater levels of impairment while working, with greater than 15 times higher odds of experiencing work impairment relative to the no LC cohort. These findings build on prior work to demonstrate the negative association with work productivity, beyond just unemployment. Moreover, these results combined with the large proportion requiring reduced work hours reinforce the need for flexible work arrangements, including remote work options, modified schedules, and supportive workplace policies, to ensure that experienced and engaged workers remain in the workforce.

Beyond the workplace, individuals with current LC also experienced significant disruptions in daily activities, reporting 20.5% impairment in their nonwork activities. This was nearly 10-fold higher than the impairment seen in those without LC. These findings are consistent with other research suggesting that the impact of LC extends beyond employment, affecting social engagement and overall quality of life.^4^

The economic burden of LC was evident in the financial toxicity scores. The current LC group had 3.16 times higher odds of moderate to high financial toxicity compared with the resolved LC cohort and 5.20 times higher odds compared with those never experiencing LC. This financial strain could be due to medical expenses, loss of employment, hour reductions due to reduced work capacity, or lower work productivity resulting in reduced bonuses or raises. The data provided here contribute to the information required by policymakers to quantify the extent of how LC financially burdens individuals at the population level.^21^ Addressing the financial burden of LC may therefore require policy interventions, such as expanded disability benefits or workplace accommodations to help combat the work and financial impact of this condition.

Importantly, we were able to demonstrate lower rates of work impairment and better financial toxicity scores in the vaccinated cohort compared with the unvaccinated cohort. This is consistent with our existing data demonstrating benefits in symptom reduction and quality of life among adults who were vaccinated for SARS-CoV-2.^4,22^ This provides further key support for the beneficial role of vaccination on patient-relevant outcomes, extending the benefit to financial and work outcomes.

### Limitations

This study has some limitations. Participant LC status was based on self-report rather than objective testing or specific symptom criteria. Consequently, this may include alternate conditions not reflective of LC. However, our approach is consistent with the most recent recommendations for defining LC, which emphasizes the myriad symptoms and importance of patient involvement with defining LC.^14^ Eligibility criteria required that participants have access to an internet-capable device, which may reflect a population with more technological access or resources. Our sample population was also more commonly non-Hispanic/Latino, White, female, and younger, which may reflect a more limited representation in the sample population compared with the US population. While we separated out the resolved LC cohort to better understand the delayed financial impact of prior LC vs never having LC, the resolved LC cohort sample was small, which led to reduced precision in the estimates, potentially requiring a larger difference to achieve statistical significance.

## Conclusions

The results of this cohort study provide critical insights into the broader associations of LC with employment, work abilities, and financial stability and the mitigating associations of prior vaccination against SARS-CoV-2. The substantial employment and economic burdens reported here underscore the need for targeted policy interventions and greater workplace support structures to ensure that the sizable US workforce that may have LC is able to contribute to economic activity and to avoid personal economic hardship. Future research should investigate potential strategies to mitigate the impact of LC, including long-term workplace policies, disability support frameworks, and tailored health care approaches for individuals affected by persistent symptoms of LC.

## Notes

Supplement 1. **eAppendix.** INSPIRE Group **eFigure 1.** Unadjusted differences in work productivity among current, resolved, and never-having Long COVID **eFigure 2.** Unadjusted difference in financial toxicity among current, resolved, and never-having Long COVID **eFigure 3.** Unadjusted difference in severity of financial toxicity among current, resolved, and never-having Long COVID **eTable 1.** Adjusted difference in outcomes between participants vaccinated and not vaccinated before the initial infection **eResults.** Outcomes by age, race, ethnicity, and gender

Supplement 2.
INSPIRE Group Members

Supplement 3.
Data Sharing Statement

## References

[1] World Health Organization. WHO COVID-19 dashboard. Accessed May 29, 2025. https://data.who.int/dashboards/covid19/cases

[2] Agency for Healthcare Research and Quality. Statistical Brief #557: prevalence of long COVID among adults who have ever had COVID-19, by selected demographic and socioeconomic characteristics, U.S. civilian noninstitutionalized population, spring 2023. September 2024. Accessed January 19, 2025. https://www.meps.ahrq.gov/data_files/publications/st557/stat557.shtml 39808055

[3] Marjenberg Z, Leng S, Tascini C, . Risk of long COVID main symptoms after SARS-CoV-2 infection: a systematic review and meta-analysis. Sci Rep. 2023;13(1):15332. doi:10.1038/s41598-023-42321-9 37714919 PMC10504382

[4] Gottlieb M, Yu H, Chen J, . Differences in long COVID severity by duration of illness, symptom evolution, and vaccination: a longitudinal cohort study from the INSPIRE Group. Lancet Reg Health Am. 2025;44:101026. doi:10.1016/j.lana.2025.101026 40040820 PMC11875141

[5] Gualano MR, Rossi MF, Borrelli I, . Returning to work and the impact of post COVID-19 condition: a systematic review. Work. 2022;73(2):405-413. doi:10.3233/WOR-220103 35938280

[6] Ottiger M, Poppele I, Sperling N, Schlesinger T, Müller K. Work ability and return-to-work of patients with post-COVID-19: a systematic review and meta-analysis. BMC Public Health. 2024;24(1):1811. doi:10.1186/s12889-024-19328-6 38973011 PMC11229229

[7] Rhead R, Wels J, Moltrecht B, . Long COVID and financial outcomes: evidence from four longitudinal population surveys. J Epidemiol Community Health. 2024;78(7):458-465. doi:10.1136/jech-2023-221059 38508701 PMC11187380

[8] Hair NL, Urban C. Association of severe COVID-19 and persistent COVID-19 symptoms with economic hardship among US families. JAMA Netw Open. 2023;6(12):e2347318. doi:10.1001/jamanetworkopen.2023.47318 38085541 PMC10716716

[9] Pisu M, Martin MY. Financial toxicity: a common problem affecting patient care and health. Nat Rev Dis Primers. 2022;8(1):7. doi:10.1038/s41572-022-00341-1 35145106 PMC9731797

[10] von Elm E, Altman DG, Egger M, Pocock SJ, Gøtzsche PC, Vandenbroucke JP; STROBE Initiative. The Strengthening the Reporting of Observational Studies in Epidemiology (STROBE) statement: guidelines for reporting observational studies. Ann Intern Med. 2007;147(8):573-577. doi:10.7326/0003-4819-147-8-200710160-00010 17938396

[11] O’Laughlin KN, Thompson M, Hota B, ; INSPIRE Investigators. Study protocol for the Innovative Support for Patients with SARS-COV-2 Infections Registry (INSPIRE): a longitudinal study of the medium and long-term sequelae of SARS-CoV-2 infection. PLoS One. 2022;17(3):e0264260. doi:10.1371/journal.pone.0264260 35239680 PMC8893622

[12] Harris PA, Taylor R, Thielke R, Payne J, Gonzalez N, Conde JG. Research Electronic Data Capture (REDCap)—a metadata-driven methodology and workflow process for providing translational research informatics support. J Biomed Inform. 2009;42(2):377-381. doi:10.1016/j.jbi.2008.08.010 18929686 PMC2700030

[13] Harris PA, Taylor R, Minor BL, ; REDCap Consortium. The REDCap consortium: building an international community of software platform partners. J Biomed Inform. 2019;95:103208. doi:10.1016/j.jbi.2019.103208 31078660 PMC7254481

[14] Committee on Examining the Working Definition for Long COVID, Board on Health Sciences Policy, Board on Global Health, Health and Medicine Division, National Academies of Sciences, Engineering, and Medicine; Fineberg HV, Brown L, Worku T, Goldowitz I, . A Long COVID Definition: A Chronic, Systemic Disease State with Profound Consequences. National Academies Press; 2024. doi:10.17226/27768 39110819

[15] Reilly MC, Zbrozek AS, Dukes EM. The validity and reproducibility of a work productivity and activity impairment instrument. Pharmacoeconomics. 1993;4(5):353-365. doi:10.2165/00019053-199304050-00006 10146874

[16] Tang K, Boonen A, Verstappen SMM, . Worker productivity outcome measures: OMERACT filter evidence and agenda for future research. J Rheumatol. 2014;41(1):165-176. doi:10.3899/jrheum.130815 24128774

[17] de Souza JA, Yap BJ, Wroblewski K, . Measuring financial toxicity as a clinically relevant patient-reported outcome: the validation of the Comprehensive Score for Financial Toxicity (COST). Cancer. 2017;123(3):476-484. doi:10.1002/cncr.30369 27716900 PMC5298039

[18] Peng X, Li G, Liu Z. Zero-inflated beta regression for differential abundance analysis with metagenomics data. J Comput Biol. 2016;23(2):102-110. doi:10.1089/cmb.2015.0157 26675626 PMC6109378

[19] Ospina R, Ferrari SLP. A general class of zero-or-one inflated beta regression models. Comput Stat Data Anal. 2012;56(6):1609-1623. doi:10.1016/j.csda.2011.10.005

[20] Dar MA, Chauhan R, Sharma KK, Trivedi V, Dhingra S, Murti K. Assessing the reliability and validity of Comprehensive Score for Financial Toxicity (COST) among radiation oncology patients in India: a cross-sectional pilot study. Ecancermedicalscience. 2021;15:1219. doi:10.3332/ecancer.2021.1219 34158823 PMC8183655

[21] Weathers RR II, Silanskis C, Stegman M, Jones J, Kalasunas S. Expanding access to health care for Social Security Disability Insurance beneficiaries: early findings from the accelerated benefits demonstration. Soc Secur Bull. 2010;70(4):25-47. 21261168

[22] Gottlieb M, Wang RC, Yu H, ; Innovative Support for Patients with SARS-CoV-2 Infections Registry (INSPIRE) Group. Severe fatigue and persistent symptoms at 3 months following severe acute respiratory syndrome coronavirus 2 infections during the pre-Delta, Delta, and Omicron time periods: a multicenter prospective cohort study. Clin Infect Dis. 2023;76(11):1930-1941. doi:10.1093/cid/ciad045 36705268 PMC10249989

