# Supplementary material for: Work Impairment and Financial Outcomes Among Adults With vs Without Long COVID
Source: JAMA Netw Open. 2025 Aug 12;8(8):e2526310. doi: 10.1001/jamanetworkopen.2025.26310 (PMC12344534; doi:10.1001/jamanetworkopen.2025.26310)
Supplement: Supplement 1. — eAppendix. INSPIRE Group eFigure 1. Unadjusted differences in work productivity among current, resolved, and never-having Long COVID eFigure 2. Unadjusted difference in financial toxicity among current, resolved, and never-having Long COVID eFigure 3. Unadjusted difference in severity of financial toxicity among current, resolved, and never-having Long COVID eTable 1. Adjusted difference in outcomes between participants vaccinated and not vaccinated before the initial infection eResults. Outcomes by age, race, ethnicity, and gender [file jamanetwopen-e2526310-s001.pdf]

Supplemental Online Content

Gottlieb M, Chen J, Yu H, et al; INSPIRE Group. Work impairment and financial outcomes among adults with vs without long COVID. *JAMA Netw Open*. 2025;8(8):e2526310. doi:10.1001/jamanetworkopen.2025.26310

eAppendix. INSPIRE Group
eFigure 1. Unadjusted differences in work productivity among current, resolved, and never-having Long COVID
eFigure 2. Unadjusted difference in financial toxicity among current, resolved, and never-having Long COVID
eFigure 3. Unadjusted difference in severity of financial toxicity among current, resolved, and never-having Long COVID
eTable 1. Adjusted difference in outcomes between participants vaccinated and not vaccinated before the initial infection
eResults.

This supplemental material has been provided by the authors to give readers additional information about their work.

© 2025 Gottlieb M et al. *JAMA Network Open*.

eAppendix.

INSPIRE Group

Rush University, Administrative Core & Enrolling Site

Study-wide Co- Principal Investigators: Robert A. Weinstein, MD, Principal Investigator; Michael Gottlieb, MD, Principal Investigator

Core research team: Michelle Santangelo, MS, Research Manager; Katherine Koo, MS-HSM, Program Manager; Antonia Derden, BA, Administrative Assistant

Site Investigators: Michael Gottlieb, MD, Site Principal Investigator

Site research team: Kristyn Gatling, MA, Research Coordinator. Research Assistants: Zohaib Ahmed, MS; Chloe Gomez; Diego Guzman, BS; Minna Hassaballa, BA; Ryan Jerger; Amro (Marshall) Kaadan, ScM

Yale University, Analytic Core & Enrolling Site

Core Investigators: Principal Investigators: Arjun Venkatesh, MD, MBA, MHS; Erica S. Spatz MD, MHS

Core research team: Research Managers: Jeremiah Kinsman, MPH, NREMT, Caitlin Malicki, MPH. Statisticians: Zhenqiu Lin, PhD; Shu-Xia Li, PhD; Huihui Yu, PhD; Imtiaz Ebna Mannan, MS; Zimo Yang, MS; Mengni Liu, MS

Site Investigators: Site Principal Investigators: Arjun Venkatesh, MD, MBA, MHS, Erica S. Spatz MD, MPH. Site Co-Investigator: Andrew Ulrich, MD

Site Research team: Research Managers: Jeremiah Kinsman, MPH, NREMT, Caitlin Malicki, MPH. Research Coordinator: Jocelyn Dorney, MPH. Research Assistants: Senyte Pierce, BA; Xavier Puente, BA; Wafa Salah, BA

© 2025 Gottlieb M et al. *JAMA Network Open*.

University of Washington, Clinical Core & Enrolling Site

Core Investigators: Graham Nichol, MD, Principal Investigator; Kari A. Stephens PhD, MS, Co-Principal Investigator

Core research team: Jill Anderson, BSN, RN, Clinical Core Program Manager; Mary Schiffgens, MBA, Grant & Finance Manager; Dana Morse, RN, BSN, Research Coordinator; Karen Adams, BA, Regulatory Specialist; Tracy Stober, BA, MA, Patient Representative; Zenoura Maat, Research Assistant

Site Investigators: Kelli N. O'Laughlin, MD, MPH, Site Principal Investigator; Nikki Gentile, MD, PhD, Co-Investigator

Site research team: Research Coordinators: Rachel E. Geyer, MPH; Michael Willis, AS, BSHS; Zihan Zhang, MS, Analyst; Gary Chang, PhD, Senior Biostatistician. Victoria Lyon, MPH, Project Manager. Research Assistants: Robin E. Klabbers, MSc in Medicine, MSc in Global Health; Luis Ruiz, BA; Kerry Malone, BA; Jasmine Park

Thomas Jefferson University, Enrolling Site

Site Investigators: Kristin Rising, MD, MS, Site Principal Investigator; Efrat Kean, MD, Co-Investigator; Anna Marie Chang, MD, MSCE

Site research team: Nurse Coordinator: Nicole Renzi, RN. Program Manager: Phillip Watts, BA, MM, CCRP. Research Coordinators: Morgan Kelly, BS; Kevin Schaeffer, BS; Dylan Grau, BS; David Cheng, BS; Carly Shutty, BSN; Alex Charlton, BS; Lindsey Shughart, BS; Hailey

© 2025 Gottlieb M et al. *JAMA Network Open*.

Shughart, BA, CCRP; Grace Amadio, MD, CCRP; Jessica Miao, BA. Research Assistants: Paavali Hannikainen, BS

University of California, Los Angeles, Enrolling Site

Site Investigators: Joann G. Elmore, MD, MPH, Site Principal Investigator, Lauren E. Wisk, PhD, Co-Investigator

Site research team: Michelle L'Hommedieu, PhD, Site Program Director; Chris Chandler, BA, Research Assistant; Megan Eguchi, MPH, Data Analyst; Kate Diaz Roldan, MPH, Research Assistant; Raul Moreno, BA, Administrative Analyst

University of California, San Francisco, Enrolling Site

Site Investigators: Robert Rodriguez, MD, Site Principal Investigator; Ralph C. Wang, MD, MAS, Site Principal Investigator; Juan Carlos Montoy, MD, PhD, Site Principal Investigator

Site research team: Robin Kemball, MPH, Program Manager; Research Coordinators: Virginia Chan, MPH; Cecilia Lara Chavez; Angela Wong, BA; Mireya Arreguin, BS

University of Texas Health Science Center at Houston, Enrolling Site

Site Investigators: Mandy J. Hill, DrPH, MPH, Site Principal Investigator; Ryan Huebinger Site, MD, Site Principal Investigator.

Site research team: Arun Kane, BA, Research Coordinator; Peter Nikonowicz, BA, Research Coordinator; Sarah Sapp, MPH, Research Coordinator

University of Texas Southwestern Medical Center, Enrolling Site

© 2025 Gottlieb M et al. *JAMA Network Open*.

Site Investigators: Ahamed H. Idris, MD, Site Principal Investigator; Samuel McDonald, MD, Co-Investigator

Site research team: David Gallegos, BS, Research Coordinator; Katherine Riley Martin, BS, MS, Research Assistant

Centers for Disease Control and Prevention (CDC)

Investigators: Sharon Saydah, PhD; Ian D. Plumb, MBBS, MSc; Aron J. Hall, DVM, MSPH; Melissa Briggs-Hagen, MD, MPH

Public Health Seattle King County: We would like to thank Public Health Seattle King County for their assistance with participant recruitment for this study.

California Department of Public Health: We would like to thank the California Department of Public Health for their assistance with participant recruitment for this study.

CTSI COVID Clinical Research Steering Committee and the CTSI Office of Clinical Research Patient Navigation Team and Bioinformatics Program: We would like to thank the CTSI COVID Clinical Research Steering Committee and the CTSI Office of Clinical Research Patient Navigation Team and Bioinformatics Program for assistance with study recruitment.

University of Washington Institute of Translational Health Sciences (ITHS): We would like to thank the ITHS for support of the REDCap instance and for biomedical informatics resources used by the UW Clinical Core and Enrolling Site to enable study recruitment, which is funded by

© 2025 Gottlieb M et al. *JAMA Network Open*.

the National Center for Advancing Translational Sciences of the National Institutes of Health under award number UL1TR002319.

© 2025 Gottlieb M et al. *JAMA Network Open*.

Supplemental Figure 1. Unadjusted differences in work productivity among current, resolved, and never-having Long COVID

Continous outcomes with excess zero values	Contrasts		Zero part (probability of having > 0 outcome)			Non-zero part (mean percent when the outcome >0)		
	Comparison	Reference	OR (95% CI)	*p*	Higher odds for Reference Group / Higher odds for Comparison Group	OR (95% CI)	*p*	Higher odds for Reference Group / Higher odds for Comparison Group
WPAI - Percent work time missed due to health	Resolved LC	Never had LC	1.99 (0.77,5.16)	0.15		0.67 (0.23,2.00)	0.48	
	Current LC	Never had LC	2.89 (2.21,3.79)	<0.0001		1.25 (0.91,1.71)	0.17	
	Current LC	Resolved LC	1.45 (0.56,3.77)	0.44		1.85 (0.62,5.53)	0.27	
WPAI - Percent impairment while working due to health	Resolved LC	Never had LC	2.90 (1.27,6.66)	0.012		0.68 (0.32,1.43)	0.31	
	Current LC	Never had LC	10.73 (8.47,13.58)	<0.0001		1.30 (1.06,1.59)	0.010	
	Current LC	Resolved LC	3.69 (1.62,8.41)	0.002		1.91 (0.92,3.99)	0.08	
WPAI - Percent overall work impairment due to health	Resolved LC	Never had LC	2.55 (1.24,5.24)	0.011		0.85 (0.45,1.60)	0.62	
	Current LC	Never had LC	7.10 (5.78,8.73)	<0.0001		1.43 (1.21,1.70)	<0.0001	
	Current LC	Resolved LC	2.79 (1.35,5.74)	0.005		1.68 (0.90,3.15)	0.10	
WPAI - Percent activity impairment due to health	Resolved LC	Never had LC	1.71 (0.72,4.09)	0.23		0.56 (0.24,1.32)	0.19	
	Current LC	Never had LC	8.36 (6.77,10.34)	<0.0001		1.43 (1.18,1.73)	0.0002	
	Current LC	Resolved LC	4.89 (2.05,11.65)	0.0003		2.54 (1.09,5.90)	0.030	

0.1 1.0 10.0 100.0 — Unadjusted Odds Ratio (95% CI) (both panels)

Supplemental Figure 2. Unadjusted difference in financial toxicity among current, resolved, and never-having Long COVID

Continous outcomes following normal distribution	Contrasts		Unadjusted LS Mean Difference		Higher LS Mean for Reference Group / Higher LS Mean for Comparison Group
	Comparison	Reference	Mean Difference (95% CI)	*p*	
Financial Toxicity (Higher is Better)	Resolved LC	Never had LC	-2.91 (-5.24,-0.58)	0.014	
	Current LC	Never had LC	-8.38 (-9.07,-7.69)	<0.0001	
	Current LC	Resolved LC	-5.47 (-7.84,-3.10)	<0.0001	

-10 -5 0 5 10 — Unadjusted LS Mean Difference (95% CI)

Supplemental Figure 3. Unadjusted difference in severity of financial toxicity among current, resolved, and never-having Long COVID

Binary outcomes	Contrasts		Unadjusted OR		Higher Odds for Reference Group / Higher Odds for Comparison Group
	Comparison	Reference	OR (95% CI)	*p*	
Financial Toxicity Moderate/high (<14) vs None/mild (≥14, Reference Group)	Resolved LC	Never had LC	1.71 (0.67,4.34)	0.26	
	Current LC	Never had LC	5.73 (4.52,7.26)	<0.0001	
	Current LC	Resolved LC	3.35 (1.33,8.45)	0.010	

0.1 1.0 10.0 — Unadjusted Odds Ratio (95% CI)

© 2025 Gottlieb M et al. *JAMA Network Open*.

Supplemental Table 1. Adjusted difference in outcomes between participants vaccinated and not vaccinated before the initial infection

**Outcomes**	**Difference between Participants vaccinated vs not vaccinated before initial infection**			
**Continuous outcomes following normal distribution**	**Adjusted LS mean Difference (95% CI)**	***p***		
Financial Toxicity (Higher is Better)	1.07 (0.19,1.95)	0.018		
**Binary outcomes**	Adjusted OR (95% CI)	*p*		
Financial Toxicity<br>Moderate/high (<14) vs None/mild (≥14, Reference group)	0.77 (0.57,1.04)	0.08		
**Continuous outcomes with excess zero values**	**Zero part<br>(probability of having > 0 outcome)**		**Non-zero part<br>(mean percent when the outcome >0)**	
	**Adjusted OR (95% CI)**	***p***	**Adjusted OR (95% CI)**	***p***
WPAI - Percent work time missed due to health	1.01 (0.70,1.46)	0.94	1.29 (0.85,1.98)	0.24
WPAI - Percent impairment while working due to health	0.66 (0.50,0.87)	0.003	0.93 (0.73,1.18)	0.54
WPAI - Percent overall work impairment due to health	0.71 (0.55,0.92)	0.01	0.91 (0.73,1.13)	0.4
WPAI - Percent activity impairment due to health	0.74 (0.57,0.96)	0.024	0.87 (0.69,1.10)	0.25

Adjustment included age, sex, race, ethnicity, and SARS-CoV-2 vaccination status before the initial infection

© 2025 Gottlieb M et al. *JAMA Network Open*.

eResults. Outcomes by Age, Race, Ethnicity, and Gender

Supplemental Table 2. Outcomes by age group.

		**Age group**				
		18 to 34	35 to 49	50 to 64	65+	p
		n=1532	n=1171	n=659	n=274	
**Outcome: financial Toxicity, higher is better (mean (SD))**		28.00 (9.57)	28.18 (10.42)	29.60 (10.70)	33.47 (9.62)	<0.001
**Outcome: financial Toxicity - absent (≥26) vs present (<26) (%)**	absent, ≥26	986 (64.4)	747 (63.8)	460 (69.8)	226 (82.5)	<0.001
	present, <26	546 (35.6)	424 (36.2)	199 (30.2)	48 (17.5)	
**Outcome: financial Toxicity - none/mild (≥14) vs moderate/high (<14) (%)**	none/mild, ≥14	1397 (91.2)	1042 (89.0)	596 (90.4)	259 (94.5)	0.027
	moderate/high, <14	135 (8.8)	129 (11.0)	63 (9.6)	15 (5.5)	
**Outcome: financial Toxicity - none, mild, moderate, and severe (%)**	Grade 0	986 (64.4)	747 (63.8)	460 (69.8)	226 (82.5)	<0.001
	Grade 1	411 (26.8)	295 (25.2)	136 (20.6)	33 (12.0)	
	Grade 2	129 (8.4)	123 (10.5)	58 (8.8)	15 (5.5)	
	Grade 3	6 (0.4)	6 (0.5)	5 (0.8)	0 (0.0)	
**Outcome: WPAI - Percent work time missed due to health (mean (SD))**		2.51 (10.96)	2.70 (12.23)	4.56 (17.57)	10.00 (27.21)	<0.001
**Outcome: WPAI - Percent impairment while working due to health (mean (SD))**		4.99 (14.43)	5.50 (15.43)	5.81 (15.96)	5.06 (15.18)	0.748
**Outcome: WPAI - Percent overall work impairment due to health (mean (SD))**		6.59 (16.88)	7.10 (17.72)	7.68 (18.80)	8.13 (19.03)	0.63
**Outcome: WPAI - Percent activity impairment due to health (mean (SD))**		5.38 (15.27)	6.43 (17.18)	6.74 (18.22)	8.16 (21.51)	0.169
**Outcome: WPAI - Percent work time missed due to health (%)**	have 0 percent	1106 (91.6)	876 (92.0)	427 (89.3)	73 (83.9)	0.032
	have >0 percent	101 (8.4)	76 (8.0)	51 (10.7)	14 (16.1)	
**Outcome: WPAI - Percent impairment while working due to health (%)**	have 0 percent	1020 (84.9)	792 (83.7)	390 (83.3)	71 (87.7)	0.673
	have >0 percent	182 (15.1)	154 (16.3)	78 (16.7)	10 (12.3)	
**Outcome: WPAI - Percent overall work impairment due to health (%)**	have 0 percent	969 (80.6)	754 (79.7)	369 (78.8)	65 (80.2)	0.867

© 2025 Gottlieb M et al. *JAMA Network Open*.

	have >0 percent	233 (19.4)	192 (20.3)	99 (21.2)	16 (19.8)	
**Outcome: WPAI - Percent activity impairment due to health (%)**	have 0 percent	1102 (83.8)	858 (82.8)	416 (81.7)	83 (84.7)	0.71
	have >0 percent	213 (16.2)	178 (17.2)	93 (18.3)	15 (15.3)	
**Outcome: WPAI - Percent work time missed due to health, limit to >0 percent population (mean (SD))**		29.99 (24.82)	33.87 (28.78)	42.75 (35.77)	62.14 (37.52)	0.001
**Outcome: WPAI - Percent impairment while working due to health, limit to >0 percent population (mean (SD))**		32.97 (21.31)	33.77 (22.58)	34.87 (22.78)	41.00 (20.25)	0.679
**Outcome: WPAI - Percent overall work impairment due to health, limit to >0 percent population (mean (SD))**		33.99 (23.22)	35.00 (23.91)	36.32 (25.19)	41.17 (21.91)	0.615
**Outcome: WPAI - Percent activity impairment due to health, limit to >0 percent population (mean (SD))**		33.24 (22.70)	37.42 (23.65)	36.88 (26.62)	53.33 (24.98)	0.01

© 2025 Gottlieb M et al. *JAMA Network Open*.

Supplemental Table 3. Outcomes by Race

		Race				
		White	Black or African American	Asian	Other/Multiple	p
		n=2438	n=281	n=499	n=335	
**Outcome: financial Toxicity, higher is better (mean (SD))**		29.88 (9.99)	23.12 (10.48)	29.19 (8.67)	25.54 (10.63)	<0.001
**Outcome: financial Toxicity - absent (≥26) vs present (<26) (%)**	absent, ≥26	1708 (70.1)	129 (45.9)	350 (70.1)	189 (56.4)	<0.001
	present, <26	730 (29.9)	152 (54.1)	149 (29.9)	146 (43.6)	
**Outcome: financial Toxicity - none/mild (≥14) vs moderate/high (<14) (%)**	none/mild, ≥14	2249 (92.2)	218 (77.6)	475 (95.2)	285 (85.1)	<0.001
	moderate/high, <14	189 (7.8)	63 (22.4)	24 (4.8)	50 (14.9)	
**Outcome: financial Toxicity - none, mild, moderate, and severe (%)**	Grade 0	1708 (70.1)	129 (45.9)	350 (70.1)	189 (56.4)	<0.001
	Grade 1	541 (22.2)	89 (31.7)	125 (25.1)	96 (28.7)	
	Grade 2	178 (7.3)	62 (22.1)	24 (4.8)	46 (13.7)	
	Grade 3	11 (0.5)	1 (0.4)	0 (0.0)	4 (1.2)	
**Outcome: WPAI - Percent work time missed due to health (mean (SD))**		2.90 (12.94)	6.62 (19.95)	1.90 (8.72)	4.81 (17.92)	<0.001
**Outcome: WPAI - Percent impairment while working due to health (mean (SD))**		4.95 (14.92)	9.94 (19.44)	3.62 (10.27)	6.06 (15.67)	<0.001
**Outcome: WPAI - Percent overall work impairment due to health (mean (SD))**		6.50 (17.30)	13.55 (22.99)	5.28 (13.45)	7.81 (18.13)	<0.001
**Outcome: WPAI - Percent activity impairment due to health (mean (SD))**		5.69 (16.48)	11.02 (21.32)	3.44 (10.11)	8.09 (19.86)	<0.001
**Outcome: WPAI - Percent work time missed due to health (%)**	have 0 percent	1691 (91.6)	153 (84.5)	361 (93.3)	219 (88.7)	0.003
	have >0 percent	155 (8.4)	28 (15.5)	26 (6.7)	28 (11.3)	
**Outcome: WPAI - Percent impairment while working due to health (%)**	have 0 percent	1577 (86.2)	128 (72.3)	327 (84.5)	193 (80.1)	<0.001
	have >0 percent	253 (13.8)	49 (27.7)	60 (15.5)	48 (19.9)	
**Outcome: WPAI - Percent overall work impairment due to health (%)**	have 0 percent	1501 (82.0)	115 (65.0)	311 (80.4)	183 (75.9)	<0.001
	have >0 percent	329 (18.0)	62 (35.0)	76 (19.6)	58 (24.1)	

© 2025 Gottlieb M et al. *JAMA Network Open*.

**Outcome: WPAI - Percent activity impairment due to health (%)**	have 0 percent	1689 (84.9)	144 (70.2)	358 (85.6)	217 (78.3)	<0.001
	have >0 percent	301 (15.1)	61 (29.8)	60 (14.4)	60 (21.7)	
**Outcome: WPAI - Percent work time missed due to health, limit to >0 percent population (mean (SD))**		34.56 (30.05)	42.79 (32.37)	28.26 (19.95)	42.40 (35.68)	0.195
**Outcome: WPAI - Percent impairment while working due to health, limit to >0 percent population (mean (SD))**		35.81 (22.50)	35.92 (20.81)	23.33 (14.92)	30.42 (22.31)	<0.001
**Outcome: WPAI - Percent overall work impairment due to health, limit to >0 percent population (mean (SD))**		36.15 (24.39)	38.69 (23.16)	26.89 (18.50)	32.45 (23.88)	0.008
**Outcome: WPAI - Percent activity impairment due to health, limit to >0 percent population (mean (SD))**		37.64 (24.36)	37.05 (23.76)	24.00 (14.87)	37.33 (27.11)	0.001

© 2025 Gottlieb M et al. *JAMA Network Open*.

Supplemental Table 4. Outcomes by Ethnicity

		Ethnicity		
		Not Hispanic	Hispanic	p
		n=3082	n=510	
**Outcome: financial Toxicity, higher is better (mean (SD))**		29.34 (9.99)	25.29 (10.56)	<0.001
**Outcome: financial Toxicity - absent (≥26) vs present (<26) (%)**	absent, ≥26	2119 (68.8)	273 (53.5)	<0.001
	present, <26	963 (31.2)	237 (46.5)	
**Outcome: financial Toxicity - none/mild (≥14) vs moderate/high (<14) (%)**	none/mild, ≥14	2824 (91.6)	431 (84.5)	<0.001
	moderate/high, <14	258 (8.4)	79 (15.5)	
**Outcome: financial Toxicity - none, mild, moderate, and severe (%)**	Grade 0	2119 (68.8)	273 (53.5)	<0.001
	Grade 1	705 (22.9)	158 (31.0)	
	Grade 2	246 (8.0)	74 (14.5)	
	Grade 3	12 (0.4)	5 (1.0)	
**Outcome: WPAI - Percent work time missed due to health (mean (SD))**		3.04 (13.31)	3.82 (14.71)	0.304
**Outcome: WPAI - Percent impairment while working due to health (mean (SD))**		5.03 (14.43)	6.96 (18.31)	0.023
**Outcome: WPAI - Percent overall work impairment due to health (mean (SD))**		6.69 (17.07)	8.82 (20.23)	0.032
**Outcome: WPAI - Percent activity impairment due to health (mean (SD))**		5.70 (16.03)	8.05 (19.60)	0.008
**Outcome: WPAI - Percent work time missed due to health (%)**	have 0 percent	2131 (91.5)	325 (88.6)	0.077
	have >0 percent	197 (8.5)	42 (11.4)	
**Outcome: WPAI - Percent impairment while working due to health (%)**	have 0 percent	1951 (84.6)	299 (82.6)	0.378
	have >0 percent	356 (15.4)	63 (17.4)	
**Outcome: WPAI - Percent overall work impairment due to health (%)**	have 0 percent	1856 (80.5)	279 (77.1)	0.155
	have >0 percent	451 (19.5)	83 (22.9)	
**Outcome: WPAI - Percent activity impairment due to health (%)**	have 0 percent	2104 (83.9)	328 (79.0)	0.017

© 2025 Gottlieb M et al. *JAMA Network Open*.

	have >0 percent	404 (16.1)	87 (21.0)	
**Outcome: WPAI - Percent work time missed due to health, limit to >0 percent population (mean (SD))**		35.98 (30.23)	33.42 (30.32)	0.619
**Outcome: WPAI - Percent impairment while working due to health, limit to >0 percent population (mean (SD))**		32.58 (21.26)	40.00 (24.69)	0.013
**Outcome: WPAI - Percent overall work impairment due to health, limit to >0 percent population (mean (SD))**		34.23 (23.40)	38.45 (25.47)	0.137
**Outcome: WPAI - Percent activity impairment due to health, limit to >0 percent population (mean (SD))**		35.37 (23.36)	38.39 (25.92)	0.284

© 2025 Gottlieb M et al. *JAMA Network Open*.

Supplemental Table 5. Outcomes by Gender

		Gender			
		Female	Male	Transgender/ Non-binary/ Other	p
		n=2429	n=1067	n=62	
**Outcome: financial Toxicity, higher is better (mean (SD))**		28.23 (10.19)	30.40 (9.73)	20.48 (10.28)	<0.001
**Outcome: financial Toxicity - absent (≥26) vs present (<26) (%)**	absent, ≥26	1561 (64.3)	783 (73.4)	19 (30.6)	<0.001
	present, <26	868 (35.7)	284 (26.6)	43 (69.4)	
**Outcome: financial Toxicity - none/mild (≥14) vs moderate/high (<14) (%)**	none/mild, ≥14	2184 (89.9)	995 (93.3)	45 (72.6)	<0.001
	moderate/high, <14	245 (10.1)	72 (6.7)	17 (27.4)	
**Outcome: financial Toxicity - none, mild, moderate, and severe (%)**	Grade 0	1561 (64.3)	783 (73.4)	19 (30.6)	<0.001
	Grade 1	623 (25.6)	212 (19.9)	26 (41.9)	
	Grade 2	235 (9.7)	67 (6.3)	16 (25.8)	
	Grade 3	10 (0.4)	5 (0.5)	1 (1.6)	
**Outcome: WPAI - Percent work time missed due to health (mean (SD))**		3.49 (14.30)	2.57 (12.31)	4.99 (14.77)	0.195
**Outcome: WPAI - Percent impairment while working due to health (mean (SD))**		5.84 (15.76)	3.55 (12.06)	13.78 (26.14)	<0.001
**Outcome: WPAI - Percent overall work impairment due to health (mean (SD))**		7.60 (18.37)	5.09 (14.47)	17.21 (29.36)	<0.001
**Outcome: WPAI - Percent activity impairment due to health (mean (SD))**		6.71 (17.38)	4.00 (13.71)	18.08 (29.57)	<0.001
**Outcome: WPAI - Percent work time missed due to health (%)**	have 0 percent	1633 (90.4)	758 (92.7)	38 (84.4)	0.05
	have >0 percent	174 (9.6)	60 (7.3)	7 (15.6)	
**Outcome: WPAI - Percent impairment while working due to health (%)**	have 0 percent	1478 (82.7)	721 (88.9)	31 (68.9)	<0.001
	have >0 percent	309 (17.3)	90 (11.1)	14 (31.1)	
**Outcome: WPAI - Percent overall work impairment due to health (%)**	have 0 percent	1403 (78.5)	681 (84.0)	30 (66.7)	<0.001

© 2025 Gottlieb M et al. *JAMA Network Open*.

	have >0 percent	384 (21.5)	130 (16.0)	15 (33.3)	
**Outcome: WPAI - Percent activity impairment due to health (%)**	have 0 percent	1593 (81.4)	787 (88.3)	32 (61.5)	<0.001
	have >0 percent	364 (18.6)	104 (11.7)	20 (38.5)	
**Outcome: WPAI - Percent work time missed due to health, limit to >0 percent population (mean (SD))**		36.25 (30.65)	35.06 (30.63)	32.10 (24.17)	0.916
**Outcome: WPAI - Percent impairment while working due to health, limit to >0 percent population (mean (SD))**		33.79 (22.22)	32.00 (20.07)	44.29 (29.28)	0.153
**Outcome: WPAI - Percent overall work impairment due to health, limit to >0 percent population (mean (SD))**		35.38 (24.24)	31.74 (21.49)	51.63 (28.38)	0.007
**Outcome: WPAI - Percent activity impairment due to health, limit to >0 percent population (mean (SD))**		36.07 (23.78)	34.23 (24.08)	47.00 (30.28)	0.096

© 2025 Gottlieb M et al. *JAMA Network Open*.
