# Supplementary material for: Work Impairment and Financial Outcomes Among Adults With vs Without Long COVID
Source: JAMA Netw Open. 2025 Aug 12;8(8):e2526310. doi: 10.1001/jamanetworkopen.2025.26310 (PMC12344534; doi:10.1001/jamanetworkopen.2025.26310)
Supplement: Supplement 2. — INSPIRE Group Members [file jamanetwopen-e2526310-s002.pdf]

Supplemental Online Content: Nonauthor Collaborators
*First name, last name, and suffix (if applicable) are required and will appear in PubMed.

*Group Name(s): INSPIRE Group

*First Name and Middle Initial(s)	*Last Name	*Suffix (eg, Jr, III)	Academic Degrees	Institution	Location (city, state/province, country)	Role or Contribution, eg, chair, principal investigator	Group (if more than 1 Group listed in the byline) and/or Subgroup (eg, Steering Committee)
Katherine	Koo		MS-HSM	Rush University	Chicago, Illinois, USA	Program Manager	INSPIRE Group
Antonia	Derden		BA	Rush University	Chicago, Illinois, USA	Administrative Assistant	INSPIRE Group
Zohaib	Ahmed		MS	Rush University	Chicago, Illinois, USA	Research Assistant	INSPIRE Group
Chloe	Gomez			Rush University	Chicago, Illinois, USA	Research Assistant	INSPIRE Group
Diego	Guzman		BS	Rush University	Chicago, Illinois, USA	Research Assistant	INSPIRE Group
Minna	Hassaballa		BA	Rush University	Chicago, Illinois, USA	Research Assistant	INSPIRE Group
Amro (Marshall)	Kaadan		ScM	Rush University	Chicago, Illinois, USA	Research Assistant	INSPIRE Group
Jeremiah	Kinsman		MPH, NREMT	Yale University	New Haven, Connecticut, U	Research Manager	INSPIRE Group
Zhenqiu	Lin		PhD	Yale University	New Haven, Connecticut, U	Statistician	INSPIRE Group
Shu-Xia	Li		PhD	Yale University	New Haven, Connecticut, U	Statistician	INSPIRE Group
Imtiaz Ebna	Mannan		MS	Yale University	New Haven, Connecticut, U	Statistician	INSPIRE Group
Zimo	Yang		MS	Yale University	New Haven, Connecticut, U	Statistician	INSPIRE Group
Mengni	Liu		MS	Yale University	New Haven, Connecticut, U	Statistician	INSPIRE Group
Andrew	Ulrich		MD	Yale University	New Haven, Connecticut, U	Site Co-Investigator	INSPIRE Group
Jocelyn	Dorney		MPH	Yale University	New Haven, Connecticut, U	Research Coordinator	INSPIRE Group
Senyte	Pierce		BA	Yale University	New Haven, Connecticut, U	Research Assistant	INSPIRE Group
Xavier	Puente		BA	Yale University	New Haven, Connecticut, U	Research Assistant	INSPIRE Group
Wafa	Salah		BA	Yale University	New Haven, Connecticut, U	Research Assistant	INSPIRE Group
Graham	Nichol		MD, MPH, FRCP	University of Washington	Seattle, Washington, USA	Principal Investigator	INSPIRE Group
Jill	Anderson		BSN, RN	University of Washington	Seattle, Washington, USA	Clinical Core Program Manage	INSPIRE Group
Mary	Schiffgens		MBA	University of Washington	Seattle, Washington, USA	Grant & Finance Manager	INSPIRE Group
Dana	Morse		RN, BSN	University of Washington	Seattle, Washington, USA	Research Coordinator	INSPIRE Group
Karen	Adams		BA	University of Washington	Seattle, Washington, USA	Regulatory Specialist	INSPIRE Group
Tracy	Stober		BA, MA	University of Washington	Seattle, Washington, USA	Patient Representative	INSPIRE Group
Zenoura	Maat			University of Washington	Seattle, Washington, USA	Research Assistant	INSPIRE Group
Michael	Willis		AS, BSHS	University of Washington	Seattle, Washington, USA	Research Coordinator	INSPIRE Group
Zihan	Zhang		MS	University of Washington	Seattle, Washington, USA	Analyst	INSPIRE Group
Gary	Chang		PhD	University of Washington	Seattle, Washington, USA	Senior Biostatistician	INSPIRE Group
Victoria	Lyon		MPH	University of Washington	Seattle, Washington, USA	Project Manager	INSPIRE Group
Robin E.	Klabbers		MSc in Medicin	University of Washington	Seattle, Washington, USA	Research Assistant	INSPIRE Group
Luis	Ruiz		BA	University of Washington	Seattle, Washington, USA	Research Assistant	INSPIRE Group
Kerry	Malone		BA	University of Washington	Seattle, Washington, USA	Research Assistant	INSPIRE Group
Jasmine	Park		BSN, RN	University of Washington	Seattle, Washington, USA	Research Assistant	INSPIRE Group
Anna Marie	Chang		MD, MSCE	Thomas Jefferson University	Philadelphia, Pennsylvania,	Co-Investigator	INSPIRE Group

Supplemental Online Content: Nonauthor Collaborators
*First name, last name, and suffix (if applicable) are required and will appear in PubMed.

***First Name and Middle Initial(s)**	***Last Name**	***Suffix (eg, Jr, III)**	Academic Degrees	Institution	Location (city, state/province, country)	Role or Contribution, eg, chair, principal investigator	Group (if more than 1 Group listed in the byline) and/or Subgroup (eg, Steering Committee)
Nicole	Renzi		RN	Thomas Jefferson University	Philadelphia, Pennsylvania,	Nurse Coordinator	INSPIRE Group
Phillip	Watts		BA, MM, CCRP	Thomas Jefferson University	Philadelphia, Pennsylvania,	Program Manager	INSPIRE Group
Morgan	Kelly		BS	Thomas Jefferson University	Philadelphia, Pennsylvania,	Research Coordinator	INSPIRE Group
Kevin	Schaeffer		BS	Thomas Jefferson University	Philadelphia, Pennsylvania,	Research Coordinator	INSPIRE Group
Dylan	Grau		BS	Thomas Jefferson University	Philadelphia, Pennsylvania,	Research Coordinator	INSPIRE Group
David	Cheng		BS	Thomas Jefferson University	Philadelphia, Pennsylvania,	Research Coordinator	INSPIRE Group
Carly	Shutty		BSN	Thomas Jefferson University	Philadelphia, Pennsylvania,	Research Coordinator	INSPIRE Group
Alex	Charlton		BS	Thomas Jefferson University	Philadelphia, Pennsylvania,	Research Coordinator	INSPIRE Group
Lindsey	Shughart		BS	Thomas Jefferson University	Philadelphia, Pennsylvania,	Research Coordinator	INSPIRE Group
Hailey	Shughart		BA, CCRP	Thomas Jefferson University	Philadelphia, Pennsylvania,	Research Coordinator	INSPIRE Group
Grace	Amadio		MD, CCRP	Thomas Jefferson University	Philadelphia, Pennsylvania,	Research Coordinator	INSPIRE Group
Jessica	Miao		BA	Thomas Jefferson University	Philadelphia, Pennsylvania,	Research Coordinator	INSPIRE Group
Paavali	Hannikainen		BS	Thomas Jefferson University	Philadelphia, Pennsylvania,	Research Assistant	INSPIRE Group
Chris	Chandler		BA	University of California, Los Angeles	Los Angeles, California, US	Research Assistant	INSPIRE Group
Megan	Eguchi		MPH	University of California, Los Angeles	Los Angeles, California, US	Data Analyst	INSPIRE Group
Kate	Diaz Roldan		MPH	University of California, Los Angeles	Los Angeles, California, US	Research Assistant	INSPIRE Group
Raul	Moreno		BA	University of California, Los Angeles	Los Angeles, California, US	Administrative Analyst	INSPIRE Group
Robin	Kemball		MPH	University of California, San Francisc	San Francisco, California, U	Program Manager	INSPIRE Group
Virginia	Chan		MPH	University of California, San Francisc	San Francisco, California, U	Research Coordinator	INSPIRE Group
Cecilia	Lara Chavez			University of California, San Francisc	San Francisco, California, U	Research Coordinator	INSPIRE Group
Angela	Wong		BA	University of California, San Francisc	San Francisco, California, U	Research Coordinator	INSPIRE Group
Mireya	Arreguin		BS	University of California, San Francisc	San Francisco, California, U	Research Coordinator	INSPIRE Group
Ryan	Huebinger		MD	University of Texas Health Science Ce	Houston, Texas, USA	Site Principal Investigator	INSPIRE Group
Arun	Kane		BA	University of Texas Health Science Ce	Houston, Texas, USA	Research Coordinator	INSPIRE Group
Peter	Nikonowicz		BA	University of Texas Health Science Ce	Houston, Texas, USA	Research Coordinator	INSPIRE Group
Sarah	Sapp		MPH	University of Texas Health Science Ce	Houston, Texas, USA	Research Coordinator	INSPIRE Group
Ahamed H.	Idris		MD	University of Texas Southwestern Me	Dallas, Texas, USA	Site Principal Investigator	INSPIRE Group
Samuel	McDonald		MD	University of Texas Southwestern Me	Dallas, Texas, USA	Co-Investigator	INSPIRE Group
David	Gallegos		BS	University of Texas Southwestern Me	Dallas, Texas, USA	Research Coordinator	INSPIRE Group
Katherine R.	Martin		BS, MS	University of Texas Southwestern Me	Dallas, Texas, USA	Research Assistant	INSPIRE Group
Sharon	Saydah		PhD	Centers for Disease Control and Prev	Atlanta, Georgia, USA	Investigator	INSPIRE Group
Ian D.	Plumb		MBBS, MSc	Centers for Disease Control and Prev	Atlanta, Georgia, USA	Investigator	INSPIRE Group
Aron J.	Hall		DVM, MSPH	Centers for Disease Control and Prev	Atlanta, Georgia, USA	Investigator	INSPIRE Group
Melissa	Briggs-Hagen		MD, MPH	Centers for Disease Control and Prev	Atlanta, Georgia, USA	Investigator	INSPIRE Group
