# Supplementary material for: Work Impairment and Financial Outcomes Among Adults With vs Without Long COVID
Source: JAMA Netw Open. 2025 Aug 12;8(8):e2526310. doi: 10.1001/jamanetworkopen.2025.26310 (PMC12344534; doi:10.1001/jamanetworkopen.2025.26310)
Supplement: Supplement 3. — Data Sharing Statement [file jamanetwopen-e2526310-s003.pdf]

Data Sharing Statement

Gottlieb. Work Impairment and Financial Outcomes Among Adults With vs Without Long COVID. *JAMA Netw Open*. Published August 12, 2025.
doi:10.1001/jamanetworkopen.2025.26310

Data
Data available: Yes
Data types: Deidentified participant data
How to access data: Deidentified data will be made available upon completion of the full study and publicly accessible through the CDC.
When available: With publication

Supporting Documents
Document types: None

Additional Information
Who can access the data: Researchers whose proposed use of the data has been approved.
Types of analyses: For any purpose.
Mechanisms of data availability: After approval of a proposal.
